# Rasch analysis of the Mini-Mental Adjustment to Cancer Scale (mini-MAC) among a heterogeneous sample of long-term cancer survivors: A cross-sectional study

**DOI:** 10.1186/1477-7525-10-55

**Published:** 2012-05-20

**Authors:** Alison Zucca, Sylvie D Lambert, Allison W Boyes, Julie F Pallant

**Affiliations:** 1 Priority Research Centre for Health Behaviour, School of Medicine & Public Health, University of Newcastle & Hunter Medical Research Institute, Newcastle, NSW, Australia; 2 Priority Research Centre for Health Behaviour School of Medicine & Public Health, University of Newcastle, Room 230A, Level 2, David Maddison Building, Callaghan, NSW, 2308, Australia; 3 Translational Cancer Research Unit, Ingham Institute for Applied Medical Research, South Western Sydney Clinical School The University of New South Wales, Liverpool, Australia; 4 Rural Health Academic Centre, University of Melbourne, 49 Graham St, Shepparton, VIC, 3630, Australia

**Keywords:** (3–10) Cancer, Psychometrics, Coping, Adjustment, Survivor, Questionnaires, Rasch

## Abstract

**Background:**

The mini-Mental Adjustment to Cancer Scale (mini-MAC) is a well-recognised, popular measure of coping in psycho-oncology and assesses five cancer-specific coping strategies. It has been suggested that these five subscales could be grouped to form the over-arching *adaptive* and *maladptive* coping subscales to facilitate the interpretation and clinical application of the scale. Despite the popularity of the mini-MAC, few studies have examined its psychometric properties among long-term cancer survivors, and further validation of the mini-MAC is needed to substantiate its use with the growing population of survivors. Therefore, this study examined the psychometric properties and dimensionality of the mini-MAC in a sample of long-term cancer survivors using Rasch analysis.

**Methods:**

RUMM 2030 was used to analyse the mini-MAC data (n=851). Separate Rasch analyses were conducted for each of the original mini-MAC subscales as well as the over-arching adaptive and maladaptive coping subscales to examine summary and individual model fit statistics, person separation index (PSI), response format, local dependency, targeting, item bias (or differential item functioning -DIF), and dimensionality.

**Results:**

For the fighting spirit, fatalism, and helplessness-hopelessness subscales, a revised three-point response format seemed more optimal than the original four-point response. To achieve model fit, items were deleted from four of the five subscales – Anxious Preoccupation items 7, 25, and 29; Cognitive Avoidance items 11 and 17; Fighting Spirit item 18; and Helplessness-Hopelessness items 16 and 20. For those subscales with sufficient items, analyses supported unidimensionality. Combining items to form the adaptive and maladaptive subscales was partially supported.

**Conclusions:**

The original five subscales required item deletion and/or rescaling to improve goodness of fit to the Rasch model. While evidence was found for overarching subscales of *adaptive* and *maladaptive* coping, extensive modifications were necessary to achieve this result. Further exploration and validation of over-arching subscales assessing *adaptive* and *maladaptive* coping is necessary with cancer survivors.

## Introduction

The number of individuals living with a history of cancer is expected to triple by 2030 [1], as 60% of newly diagnosed patients survive for five or more years [2,3]. Cancer survivors are defined as anyone diagnosed with cancer, from the time of diagnosis to the end of life [4]. This paper focuses on long-term cancer survivors (more than 5 years post-diagnosis [5]) who often continue to face a range of daily challenges as a result of their cancer and treatment including fatigue, fear of recurrence, and employment challenges [e.g. [6-10]]. Recognition of these cancer survivorship issues has gained momentum in the last decade, and the recommendation for further research aimed at enhancing survivors’ quality of life and quality of care has been emphasized [11,12]. It is essential that such research is underpinned by robust measures.

Coping is a popular concept that is inherently attractive to clinicians and researchers alike as it offers a means for enhancing cancer survivors’ quality of life [13,14]. Coping styles are a person’s cognitive or behavioural efforts to manage the demands of a stressful situation [15,16]. Although the effectiveness of coping strategies used by individuals diagnosed with cancer varies across situations and should not be assumed a-priori, adaptive coping strategies (e.g., fighting spirit, positive focus, seeking support) are generally associated with optimal adjustment, whereas maladaptive strategies (e.g., helplessness-hopelessness, anxious preoccupation) are associated with poor psychosocial outcomes [17-19].

The Mental Adjustment to Cancer (MAC) scale is one of the most widely used instruments to measure coping responses in individuals with cancer [20]. The 29-item mini-MAC is a refinement of the original MAC scale, and its brevity allows it to be included within a battery of measures or used in clinical settings. The mini-MAC assesses five cognitive coping responses: helplessness-hopelessness (e.g., ‘*I feel like giving up*’), anxious preoccupation (e.g., ‘*I am apprehensive*’), cognitive avoidance (e.g., ‘*Not thinking about it helps me cope*’), fatalism (e.g., ‘*At the moment I take one day at a time*’), and fighting spirit (e.g., ‘*I see my illness as a challenge*’). A number of studies examining the psychometric properties of the mini-MAC have supported the reliability of all five subscales [21-29]. However, studies have also proposed that some of the subscales can be combined to form more general coping subscales. For example, Ho et al. [22] and Kang et al. [23] suggested that fatalism and fighting spirit could be combined to form a ‘positive attitude’ subscale, and Kang et al. [23] proposed that helplessness-hopelessness and anxious preoccupation could be combined to form a ‘negative emotion’ subscale. Anagnostopoulos et al. [24] proposed the ‘adaptive’ (fighting spirit, cognitive avoidance, and fatalism subscales) and ‘maladaptive’ (helplessness-hopelessness and anxious preoccupation subscales) subscales.

Whilst Ho et al. [22] and Kang et al. [23] each identified a revised set of four coping factors that somewhat varied from Watson’s original five coping factors, Anagnostopoulos et al.’s [24] study is the first to identify that the original five coping styles can exist at one level and also group together into two larger secondary factors. From a practical perspective, we were interested in exploring a structure that would allow scoring of specific coping styles, as well as overall scores on global adjustment [25]. The ability to simplify the structure of the mini-MAC from five to two dimensions may prove useful in some contexts, such as when trying to predict well-being [26] and help to facilitate its interpretation and clinical application.

Classical test theory has traditionally been used to assess a scale’s construct validity, but emerging alternative techniques such as Rasch analysis and structural equation modelling are increasingly being adopted. While structural equation modelling has been previously used with the mini-MAC [24], to our knowledge Rasch analysis has not been applied to the scale. Rasch measurement models can examine the psychometric properties of an instrument using techniques such as threshold mapping (i.e., patients with a high measure of an attribute consistently endorse high scoring response options across all items), model fit (i.e., hierarchical ordering of items is consistent over all levels of the construct) and dimensionality (i.e., whether items of a proposed scale measure a single underlying construct) [27,28].

As the number of people living with the effects of cancer increases, it is important to accurately measure their coping responses. To date, the mini-MAC has only been tested among individuals recently diagnosed with cancer or breast cancer survivors, and, to our knowledge, this is the first Rasch analysis of the mini-MAC in a large sample of long-term cancer survivors. Specifically, this study aimed to 1) assess the psychometric properties of the original factor structure of the mini-MAC among long-term cancer survivors and 2) determine whether combining subscales to form the overarching subscales of *adaptive* and *maladaptive* coping is appropriate.

## Methods

This research was a secondary analysis of the New South Wales (NSW) Cancer Survival Study, a population-based, cross-sectional study of the physical and psychosocial well-being of long-term cancer survivors [29,30]. The Human Research Ethics Committees of the University of Newcastle and Cancer Council NSW approved the study.

### Sample

Participants were randomly selected cases from the state-based (NSW) Central Cancer Registry in Australia. Participant eligibility criteria included being (1) diagnosed with a new histologically confirmed cancer (local or metastatic) five to six years ago, (2) aged between 18 and 75 years at the time of diagnosis, (3) living in NSW, and (4) considered by their clinician to be a) able to read and understand English adequately, b) physically, and mentally capable of participating, and c) aware of their cancer diagnosis. The primary treating clinician of each potentially eligible survivor was contacted by the Registry and asked to provide consent for the nominated survivor to be contacted about the study. These participants were then approached by the Registry requesting permission to provide their contact details to the researchers. The Registry provided the researchers with the contact details of the survivors who agreed to be contacted about the study.

### Data collection

Data collection occurred between April 2002 and October 2003. Survivors who agreed to being contacted about the study were mailed a pen-and-paper survey with a reply paid envelope for its return. The survey consisted of a series of instruments measuring survivors’ physical, psychological, and social well-being. Survivors who did not respond to the initial survey received a reminder survey after three weeks and a reminder telephone call three weeks thereafter. Return of the completed survey to the researchers indicated voluntary consent to participate.

Demographic and clinical characteristics including age, sex, cancer type, and spread of disease were collected from the Registry. Self-report survey items assessed the number of adults and children residing with the survivor, gross family income, current work status, highest educational qualification, marital status, health insurance status, remission status, treatments ever received and in last month, time since last hospital admission to receive cancer treatment, and treatment for psychiatric illness.

The 29-item mini-MAC was administered to assess five cancer specific coping strategies: 1) helplessness-hopelessness (8 items), 2) anxious preoccupation (8 items), 3) fighting spirit (4 items), 4) cognitive avoidance (4 items), and 5) fatalism (5 items). Each item is rated on a 4-point scale ranging from 1=‘Definitely does not apply to me’ to 4=‘Definitely applies to me’. A higher subscale score indicates stronger use of the coping strategy. The mini-MAC has demonstrated reliability with Cronbach’s alpha coefficients for each domain ranging from 0.62–0.88 [17]. The mini-MAC does not distinguish between state- and trait-like coping responses.

### Data Analysis

Rasch analysis is a modern and rigorous psychometric approach increasingly used to obtain in-depth understanding of a scale’s measurement properties [27,31], and to identify measurement issues not easily detected by traditional analyses (e.g., item bias, response format) [31]. Rasch analysis involves testing of a scale against a mathematical measurement model developed by the Danish mathematician Georg Rasch [32]. The Rasch measurement model assumes that the probability of a participant endorsing an item is a logistic function of the relative difference between the item’s location (difficulty of the item) and the person’s location (ability of the person). The mathematical Rasch model is considered the formal representation of ‘proper’ measurement against which data are examined. Hence, the overall objective of the analysis is to test the extent to which the observed pattern of item responses conforms to Rasch model expectations [33,34]. The Rasch procedures and guidelines used in this analysis are consistent with those recommended by Pallant and Tennant [28] and Tennant and Conaghan [27] and other analyses conducted by our team [34].

The initial step in Rasch analysis is to decide which mathematical derivation of the Rasch model should be chosen. When items have three or more options, as in the case of the mini-MAC, one of two Rasch models need to be chosen –the Rating Scale Model [35] or the Partial Credit Model [36]. The principal difference between these two models is that the Rating Scale Model expects the distance between thresholds (*threshold* refers to the point between two response categories where either response is equally probable) to be equal across items. That is, that the metric distance between the thresholds separating categories 1 and 2, for example, and the ones separating categories 2 and 3 are the same across *all* items. To determine which model to use a likelihood ratio test was conducted in RUMM for each subscale. The likelihood-ratio test assessed how many times more likely the data are under the Rating Scale Model than the Partial Credit Scale Model. If the p-value of the test is not significant, then the Rating Scale model can be adopted for the analysis. In the present analysis, the p-value of the likelihood ratio test was significant for all subscales (p<.001), indicating that the distances between thresholds varies across items and it is more appropriate to use the Partial Credit model than the Rating Scale model.

The mini-MAC was analysed in two stages. Only participants with responses to all items in a given subscale were included in the analyses. First, the original, five mini-MAC subscales were analysed separately and then the appropriateness of using the broader subscales of Adaptive (cognitive avoidance, fighting spirit, and fatalism subscales combined) and Maladaptive (helplessness-hopelessness and anxious preoccupation subscales combined) coping was examined. For all subscales there was an assessment of 1) overall model fit, 2) person separation index, 3) individual item and person fit residual standard deviation (SD), 4) response format (threshold maps), 5) local dependency, 6) targeting (person-item threshold maps), 7) differential item functioning (DIF), and 8) dimensionality.

The overall fit of the scale was evaluated using chi-square statistics and the summary items and persons fit residual mean values and SDs [27,28]. As an indication of good fit, it was expected that the chi-square probability value would be non-significant (using Bonferroni alpha value adjusted to the number of items). At the summary level, a perfect fit for items and persons is represented by a mean of zero and a SD of + 1. A maximum value of 1.5 was accepted and indicative of good fit [37]. Given the sensitivity of the chi-square statistics to large sample sizes [38] (in this case n=851), the residual statistics were used primarily to guide decision-making concerning fit.

The Person Separation Index (PSI) provides an indication of the internal consistency of the scale and the power of the measure to discriminate amongst respondents with different levels of the trait being measured. The PSI is interpreted in a comparable way to Cronbach's alpha coefficient where 0.70 is considered a minimal value for group or research use and 0.85 for individual or clinical use [27].

Individual item and person fit residual values were also inspected to identify items and/or persons that might be contributing to misfit (i.e., values outside the range ± 2.5). High positive fit residual values indicate misfit, while high negative fit residuals suggest item redundancy.

Threshold maps were examined to identify disordered thresholds. When individuals do not use the response categories in a manner that is consistent with the level of the trait being measured this often results in *disordered thresholds*. If a disordered threshold was detected, item rescoring was considered, informed by the item’s category probability curve.

The presence of local dependency was also investigated. Local independence means that the response to any item is unrelated to any other item when the level of the underlying construct is controlled for. To identify local dependency, the residual correlation matrix generated in RUMM was examined and pairs of items with correlations exceeding 0.3 were taken to indicate dependency. If local dependency was detected, sub-test analysis was performed to examine whether this level of correlation artificially inflated the reliability of the subscale.

It is important, particularly in clinical practice, that a measure is well-targeted [33]. Comparison of the mean location score obtained for persons with that of the value of zero set for the items provides an indication of how well targeted the items are for the individuals in the sample [27]. It was expected that for a well-targeted measure (i.e., not too easy, not too hard), the mean location for persons, as indicated by the person-item threshold distribution maps, would be around zero. A negative mean value indicates that the sample as a whole was located at a lower level than the average scale (floor effect), while a positive value would suggest the opposite (ceiling effect) [27,37].

Potential item bias (i.e., DIF) can occur when different groups within the sample, despite equal levels of the underlying characteristic being measured, respond in a different manner to an individual item [28]. When one group shows a consistent difference in their responses to an item, across the whole range of the attribute being measured, this is referred to as *uniform DIF.* When there is non-uniformity in the differences between the group, or DIF varies across levels of the attribute, this is referred to as *non-uniform DIF*. Every item was examined for DIF across three subgroups within the sample (referred to as ‘person factors’ in RUMM) - age (four groups: 18–49, 50–59, 60–69, 70 and older), sex (male, female), and cancer type (breast, prostate, or colorectal cancer or melanoma or other). For the purpose of this analysis, the small sub-group of individuals with head and neck cancer was excluded (n=30). To assess DIF in RUMM, analysis of variance (ANOVA) of the standardized response residuals for each item was conducted across each level of the factors and class interval (i.e., at different levels of trait). A Bonferroni adjusted alpha level was then used to determine statistical significance. In addition, the importance of DIF was judged graphically. When an item was found to exhibit DIF (statistically and graphically), deletion was considered, particularly if removal improved overall model fit [39].

Last, if the subscale included enough items (i.e., more than three), dimensionality analyses were conducted. To examine dimensionality of the subscales, principal component analysis (PCA) of the residuals was performed to identify the two subsets of items that showed the most difference from one another (i.e., identify the positively and negatively loading items). Person estimates (location values) derived from the highest positive set of items were compared for each person in the sample against those derived from the highest negative set using t-tests. The number of significant t-tests, outside the + 1.96 range, indicates whether the scale is unidimensional or not. If more than 5% of these tests are significant (or specifically the lower bound of the binomial confidence interval is above 5%), the scale is multidimensional [27]. This approach has been shown to be robust to simulated levels of multidimensionality in polytomous scales [40].

To conduct the above analyses, mini-MAC data were entered into SPSS19.0 [41] and then exported into RUMM2030 [42]. In this study, 851 (out of the possible 863) survivors were included, which is adequate for the Rasch analyses conducted [43].

## Results

### Sample

A total of 863 long-term survivors consented to participate and returned a completed survey (63% participation rate). The characteristics of the sample are reported elsewhere [23,24]. In summary, just over half the sample were women, and almost three quarters were aged 50 years or over at diagnosis (range, 18 years-74 years). The majority of the sample was married or living as married and had completed secondary school. The vast majority of the sample had one of the four most incident cancer types in Australia (breast, 29%; prostate, 15%; melanoma, 15% and large bowel, 13%) and were diagnosed with a localised cancer. Although almost all (99%) of the sample had received treatment for their cancer, only 38 (5%) had received any form of active treatment for their cancer in the last month.

### Analysis of the five mini-MAC subscales

Rasch analysis was undertaken on the items in each of the five mini-MAC subscales (see Table 1 for details of the mini-MAC items, original mini-MAC subscales, and the proportion of survivors endorsing each item).

**Table 1:** Proportion of survivors endorsing each mini-MAC item

**Item number**	**Item description**	**Original subscale**	**Applies or definitely applies n (%)**
1	Take one day at a time	Fatalism	358 (42%)
2	Illness as a challenge	Fighting spirit	374 (44%)
3	Hands of God	Fatalism	405 (48%)
4	Giving up	Helplessness-hopelessness	29 (3%)
4	Angry	Anxious preoccupation	97 (11%)
6	At a loss	Helplessness-hopelessness	40 (5%)
7	Devastating feeling	Anxious preoccupation	167 (20%)
8	Count blessings	Fatalism	664 (79%)
9	Worry cancer worse	Anxious preoccupation	438 (52%)
10	Fight illness	Fighting spirit	454 (54%)
11	Distract	Cognitive avoidance	406 (48%)
12	Can't handle it	Helplessness-hopelessness	52 (6%)
13	Apprehensive	Anxious preoccupation	191 (23%)
14	Not hopeful	Helplessness-hopelessness	111 (13%)
15	Nothing to help myself	Helplessness-hopelessness	44 (5%)
16	End of the world	Helplessness-hopelessness	23 (3%)
17	Not thinking helps to cope	Cognitive avoidance	303 (36%)
18	Optimistic	Fighting spirit	674 (80%)
19	Bonus	Fatalism	548 (65%)
20	Life hopeless	Helplessness-hopelessness	23 (3%)
21	Can't cope	Helplessness-hopelessness	23 (3%)
22	Upset	Anxious preoccupation	250 (30%)
23	Beat disease	Fighting spirit	632 (75%)
24	Life is precious	Fatalism	687 (81%)
25	Belief difficult	Anxious preoccupation	71 (8%)
26	Positive effort not to think	Cognitive avoidance	492 (58%)
27	Push thoughts out of my mind	Cognitive avoidance	385 (48%)
28	Anxiety	Anxious preoccupation	94 (11%)
29	Frightened	Anxious preoccupation	313 (37%)

#### Anxious preoccupation

Analysis of the eight anxious preoccupation items revealed misfit to the Rasch model expectations, as indicated by a significant item-trait interaction chi-square probability value (p < .001) and a high item fit residual SD (SD=2.61) (Analysis 1 Table 2). No disordered thresholds were detected, providing support for the response format. High item fit residual values were noted for items 7 ‘*it is a devastating feeling*’ (2.86) and 25 ‘*difficulty in believing that this happened*’ (4.46). Different item deletions were examined to improve fit (Analyses 2–3 Table 2) with the optimal solution obtained with the deletion of items 7 and 25 (Analysis 4 Table 2). No DIF for age was found; however, DIF by sex and cancer type were evident for item 29 ‘*I am a little frightened*’ and as the deletion improved model fit, it was removed (Analysis 5 Table 2). Once item 29 was deleted, no DIF or local dependency was detected and there was no evidence of multidimensionality (Analysis 5 Table 2). The PSI was low (.71) (Analysis 5 Table 2), but is likely to have been affected by the skewed distribution (average mean person location was −1.52; SD= 1.53).

**Table 2:** Model Fit Statistics for the five mini-MAC subscales

**Action**	**Analysis**	**# of items in the subscale**	**Chi-square**		**Items Fit Residual Mean (SD)**	**Persons Fit Residual Mean (SD)**	**PSI**	**Unidimensional t-test**
			**Value**	**p**				
**Anxious Preoccupation (n= 693)**								
** Original**	1	8	191.70	< .001	.22 (2.61)	-.38 (1.20)	.80	5.57
** Item 25 removed**	2	7	147.87	< .001	-.006 (1.92)	-.39 (1.13)	.78	-
** Item 7 removed**	3	7	183.81	< .001	.07 (2.49)	-.38 (1.14)	.79	-
Items 7 and 25 removed*	4	6	137.78	< .001	-.15 (1.53)	-.39 (1.04)	.77	3.71
Items 7, 25, and 29 removed*	5	5	104.66	< .001	-.19 (1.36)	−39 (.99)	.71	3.47
**Cognitive Avoidance (n= 689)**								
** Original**	6	4	93.46	< .001	1.10 (1.85)	-.58 (1.34)	.70	3.38
** Item 11 removed**	7	3	75.91	< .001	1.20 (1.83)	-.49 ( − 1.02)	.66	-
Items 11 and 17 removed*	8	2	35.20	< .001	.30 (.32)	-.58 (.77)	.66	-
**Fighting Spirit (n=723)**								
** Original**	9	4	168.39	< .001	.31 (2.11)	-.45 (1.28)	.61	.72
** Rescoring all items**	10	4	121.66	< .001	.90 (2.51)	-.22 (.89)	.52	-
** Rescoring all items and item 18 removed**	11	3	80.43	< .001	1.23 (.94)	-.17 (.71)	.42	-
**Fatalism (n=749)**								
** Original**	12	5	106.89	< .001	.96 (.67)	-.28 (1.06)	.61	2.22
** Rescoring all items**	13	5	88.44	< .001	1.15 (.56)	-.18 (.98)	.57	.62
**Helplessness-Hopelessness (n= 437)**								
** Original**	14	8	202.20	< .001	-.21 (2.95)	-.54 (1.33)	.57	1.47
** Rescoring all items**	15	8	169.04	< .001	-.44 (2.93)	-.50 (1.35)	.59	-
** Rescoring all items and item 12 removed**	16	7	153.30	< .001	-.44 (2.82)	-.47 (1.22)	.56	-
Rescoring all items and items 12 and 14 removed*	17	6	79.59	< .001	-.55 (2.27)	-.66 (1.63)	.43	-
Rescoring all items and items 12, 14, and 20 removed*	18	5	45.00	< .001	-.05 (1.40)	-.59 (1.49)	.35	-
** Rescoring all items and item 20 removed**	19	7	136.82	< .001	-.11 (2.23)	-.46 (1.28)	.54	-
Rescoring all items and items 20 and 16 removed*	20	6	94.66	< .001	.14 (1.52)	-.45 (1.28)	.49	.74

*Items listed in the order they were deleted. Anxious preoccupation items – 5,7,9,13,22,25,28,29; Cognitive avoidance – 11,17,26,27; Fighting spirit – 2,10,18,23; Fatalism – 1,3,8,19,24; Helplessness-hopelessness – 4,6,12,14,15,16,20,21.

#### Cognitive Avoidance

**Figure 1:**
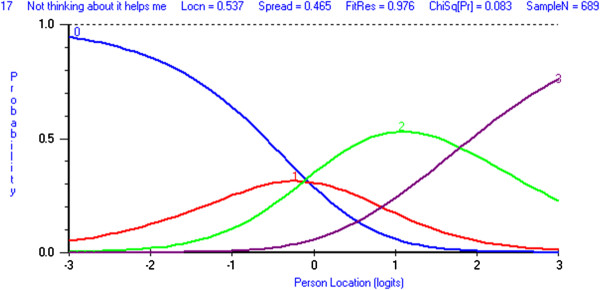
**Category Probability Curve for Cognitive Avoidance item 17*‘Not thinking about it helps me cope’.***

Analysis of the four cognitive avoidance items revealed initial misfit to the Rasch (Analysis 6 Table 2). Item 17 ‘*not thinking about it helps me cope*’ was the only item with a disordered threshold and as this disordering was minimal, no further action was taken (Figure 1). Item 11 ‘*distract myself when thoughts about my illness come into my head*’ recorded the highest individual fit residual value (3.67) and it was subsequently removed from the subscale. Overall fit was still poor (Analysis 7 Table 2), with the individual fit statistics indicating poor fit to the model for item 17 (fit residual = 3.31). Once both items 11 and 17 were removed, overall fit was acceptable and no local dependency was evident (Analysis 8 Table 2). No DIF for age or cancer type was detected; however, some items were found to have DIF for gender. At equivalent levels of cognitive avoidance, women were more likely to endorse item 26 ‘*positive effort not to think about my illness*’, whereas men were more likely to endorse item 27 ‘*push all thoughts of cancer out of my mind*’. No further action was taken, as the level of DIF is likely to cancel out at the subscale level [39]. Multidimensionality was not tested, as only two items remained in the subscale. Although the PSI was low (0.66), the subscale was reasonably well-targeted (average mean person location was −0.09, SD= 2.15).

#### Fighting Spirit

The summary item fit residual SD value for the fighting spirit items indicated misfitting items (SD= 2.11) (Analysis 9 Table 2). All items showed disordered thresholds, suggesting a problem with the response format, despite items showing an adequate number of cases in each response category (range=52 to 286). Rescoring by collapsing categories 1 ‘*definitely does not apply to me*’ and 2 ‘*does not apply to me*’ resulted in ordered thresholds for all items (Analysis 10 Table 2).

Following rescoring, item 18 ‘*I am very optimistic*’ still showed a high fit residual SD value (4.31) and was therefore removed from the scale (Analysis 11 Table 2). The overall items fit residual SD was acceptable and no local dependency or significant DIF by age, cancer type, and sex were found. Multidimensionality was not tested, as only three items remained. Given the small number of items, the PSI was well below the accepted value (0.42); however, scores were reasonably well-targeted (average mean person location was −0.35, SD= 1.41).

#### Fatalism

**Figure 2:**
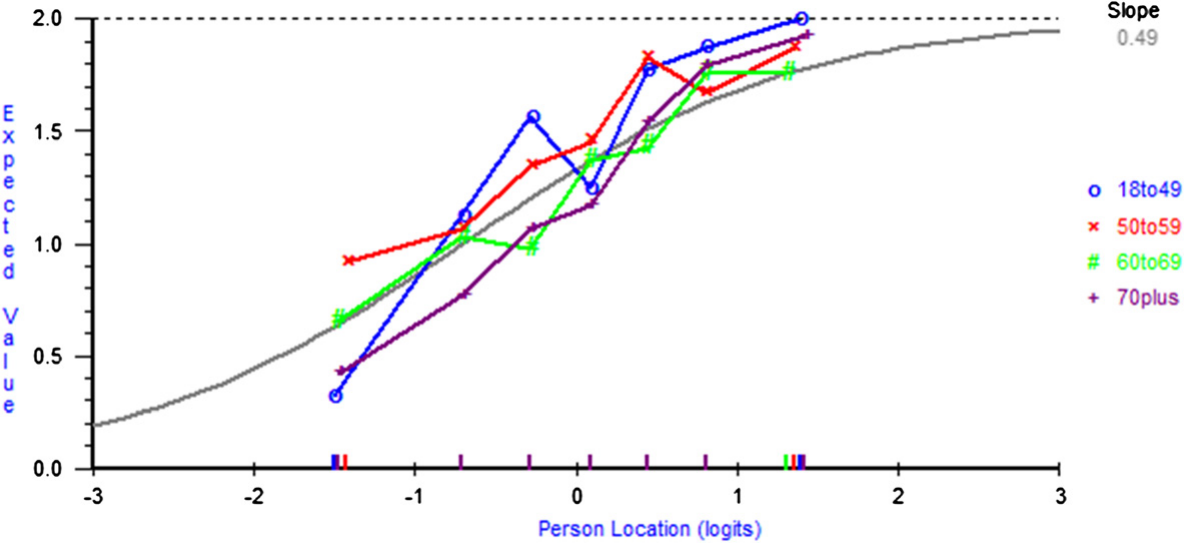
**DIF by age for item 24 ‘*I now realise how precious life is and I’m making the most of it’.***

**Figure 3:**
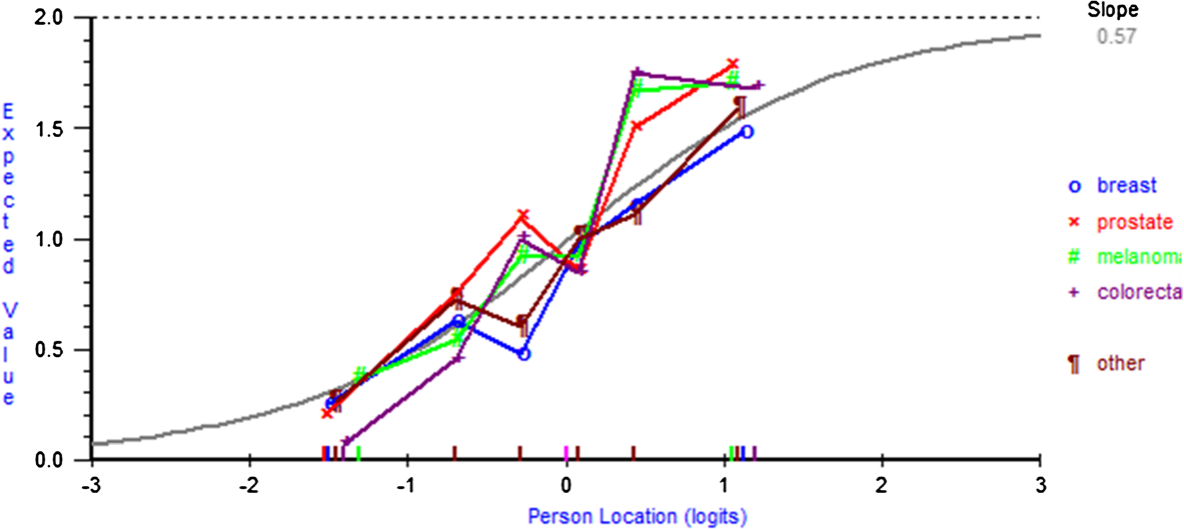
**DIF by cancer type for item 19 ‘*I’ve had a good life what’s left is a bonus*’.**

The fit residual SDs value for the fatalism subscale was within the accepted range (Analysis 12 Table 2). Although there was a reasonable number of participants across response categories (range = 51–331), all five items showed disordered thresholds and were rescored by collapsing response categories 1 ‘*definitely does not apply to me*’ and 2 ‘*does not apply to me*’ (Analysis 13 Table 2). All fit indices were acceptable; however, the PSI was low (0.57). The average mean person location was −0.13 (SD= 1.20), which suggests that overall the subscale was well-targeted. DIF by age was found for items 8 ‘*I count my blessings*’, 19 ‘*I’ve had a good life what’s left is a bonus*’, and 24 ‘*I now realise how precious life is and I’m making the most of it*’. At equivalent levels of Fatalism younger participants were more likely to endorse item 8, whereas older participants were more likely to endorse item 19. A non-uniform DIF by age for item 24 was found and a non-uniform DIF for item 19 was found for cancer type. However, as for both of these items there was no clear or meaningful interpretation of the DIF (Figures 2 and 3) and deletion did not change the interpretation of the fit statistics, no further action was taken. DIF by sex was found for two items – at equivalent levels of Fatalism women were more likely to endorse item 8, particularly at lower trait levels; however, men were more likely to endorse item 19. No further action was taken for DIF, as the level of DIF is likely to cancel out at the subscale level [39]. There was no local dependency and no evidence of multidimensionality (Analysis 13 Table 2).

#### Helplessness-Hopelessness

The residual SD value for the helplessness-hopelessness items was 2.95, suggesting the presence of misfitting items (Analysis 14 Table 2). All items showed disordered thresholds and were rescored by collapsing response categories 3 ‘*applies to me*’ and 4 ‘*definitely applies to me*’. This disorder was not due to low response frequencies, as there were sufficient cases in each response category for Rasch analysis (range=3–283). Despite rescoring, the item fit residual SD was still high (2.93) (Analysis 15 Table 2). A number of alternative solutions were considered (Analyses 16–19 Table 2) and most promising results (i.e., appropriate fit and fewer item deletions) were obtained when item 20 *‘I feel that life is hopeless*’ followed by item 16 ‘*think it is the end of the world*’ were removed (Analysis 20 Table 2). No local dependency or DIF for age, sex, or cancer type were detected and there was no evidence of multidimensionality (Analysis 20 Table 2). The PSI was low (.49), but this fit statistic is likely to have been affected by the skewed distribution (average mean person location was −1.54; SD= 1.67).

### Analysis of the Adaptive and Maladaptive Coping Subscales

#### Adaptive subscale

The Adaptive subscale, as proposed by Anagnostopoulos et al. [24], is composed of the original fighting spirit, cognitive avoidance, and fatalism items. Rasch analysis, conducted on these combined sets of items (with the rescoring and deletions detailed in the previous findings section), revealed poor model fit (Analysis 1 Table 2). A number of alternative solutions were considered (Analyses 2–4 Table 3) and to achieve item fit it was necessary to delete three of the five fatalisms items: 3 ‘*I've put myself in the hands of God*’, 8 ‘*I count my blessings*’, and 19 ‘*I've had a good life; what's left is a bonus*’ (Analysis 5 Table 3). Although the fit residual (2.66) for item 1 ‘*At the moment I take one day at a time*’ was marginally above the recommended cut point (2.5) it was retained, as the overall items fit residual SD was satisfactory. Local dependency between items 26 ‘*I make a positive effort not to think about my illness*’ and 27 ‘*I deliberately push all thoughts of cancer out of my mind*’ was detected with a residual correlation of r = 0.38. Although sub-test analysis revealed that PSI decreased from 0.74 to 0.68, overall the interpretation of the fit statistics were the same (Analysis 6 Table 3). To maintain the integrity of the scale, no further action was taken. No DIF was detected. A series of t-tests performed on the person estimates from two subsets of items identified from principal component analysis of the residuals revealed that only 3.27% of cases had statistically significant t-values (Analysis 5 Table 3). Although the PSI was low (0.74), the subscale was reasonably well-targeted (average mean person location was −0.26, SD= 1.29).

**Table 3:** Model Fit Statistics for the five mini-MAC subscales

**Action**	**Analysis**	**# of items**	**Chi-square**		**Items Fit Residual Mean (SD)**	**Persons Fit Residual Mean (SD)**	**PSI**	**Unidimensional t-test**
			**Value**	**p**				
**Adaptive (n=751)**								
Fighting spirit and Fatalism items rescored and remove Fatalism items 11 and 17 and Fighting spirit item 18* ±	1	10	165.76	< .001	.81 (1.92)	-.24 (1.35)	.79	4.35
** Remove items 11, 17, 18, and 3**	2	9	142.43	< .001	.79 (1.66)	-.27 (1.31)	.78	-
** Remove items 11, 17, 18, and 19**	3	9	170.05	< .001	.72 (2.05)	-.23 (1.26)	.77	
** Remove items 11, 17, 18, 3, and 8-**	4	8	127.12	< .001	.92 (1.42)	-.27 (1.25)	.76	--
Remove items 11, 17, 18, 3, 8, and 19*£	5	7	112.47	< .001	.78 (1.18)	-.27 (1.15)	.74	3.27
** Remove items 11, 17, 18, 3, 8, and 19 (subtest)**	6	7	166.17	< .001	.84 (.91)	-.21 (1.02)	.68	2.64
**Maladaptive (n=682)**								
Rescore all Hopelessness-helplessness items and remove Hopelessness-helplessness items 16 and 20* and Anxious preoccupation items 7,25, and 29±	7	11	313.81	< .001	-.42 (2.63)	-.44 (1.21)	.80	4.38
Remove items 16, 20, 7, 25, and 29 as well as 9, 22, 21, and 6*	8	7	123.64	< .001	.44 (0.96)	-.38 (1.18)	.63	1.25
Remove items 16, 20, 7, 25, and 29 as well as items 21, 9, 22, and 6*	9	7	123.64	< .001	.44 (0.96)	-.38 (1.18)	.63	1.25

*Items listed in the order they were deleted; **±** modifications suggested when subscales analysed separately. £ Same results if delete item 19 first followed by items 3 and 8. Original adaptive subscale 13 items = Cognitive avoidance (11,17,26,27); Fighting spirit (2,10,18,23) and Fatalism (1,3,8,19,24). Original maladaptive subscale 16 items= Helplessness-hopelessness (4,6,12,14,15,16,20,21) and Anxious preoccupation (5,7,9,13,22,25,28,29).

In summary, to fit the original 13 Adaptive items to the Rasch model expectations it was necessary to: a) rescore fatalism items (1, 3, 8, 19, and 24) and fighting spirit items (2, 10, 18, and 23) and b) remove items 11, 17, 18 as well as items 3, 8, and 19. Hence, findings suggest that the remaining fighting spirit (rescored items 2, 10, and 23), cognitive avoidance (original items 26 and 27), and fatalism (rescored items 1 and 24) items can be combined to form an Adaptive coping subscale.

#### Maladaptive subscale

**Figure 4:**
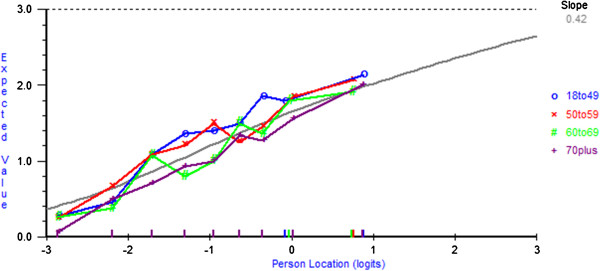
**DIF by age for item 13*‘I am apprehensive*’.**

**Figure 5:**
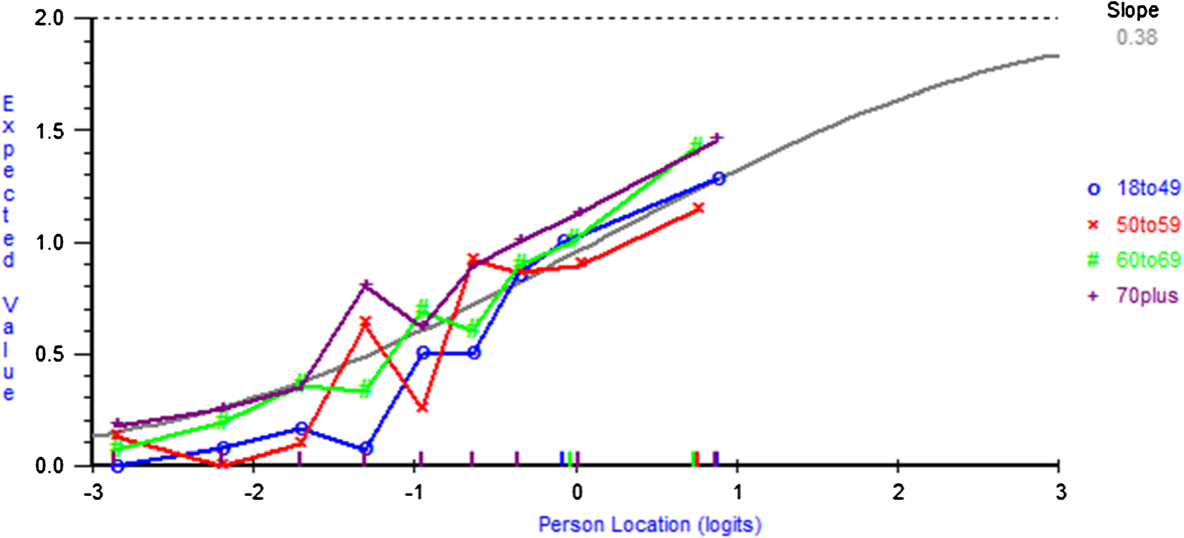
**DIF by age for item 15 ‘*I feel there is nothing I can do to help myself*’.**

The Maladaptive subscale, as proposed by Anagnostopoulos et al. [24], is composed of the original anxious preoccupation and the helplessness-hopelessness items. When the anxious preoccupation and helplessness-hopelessness subscales are revised according to the item rescoring and deletions identified in the previous findings section, the high item fit residual SD (2.63) suggests misfit (Analysis 7 Table 3). Regardless of the sequence in which items were deleted (Analysis 8 or 9 Table 3), to achieve model, fit it was necessary to remove items 9 (anxious preoccupation), 21 (helplessness-hopelessness), 22 (anxious preoccupation), and 6 (helplessness-hopelessness). No local dependency was found. DIF for sex was detected for items 13 *‘I am apprehensive*’ and 15 ‘*I feel there is nothing I can do to help myself*’. For item 13, at similar levels of the trait, older participants were less likely to endorse the item than younger individuals (Figure 4), whereas the opposite was found for item 15 (Figure 5). Hence, no further action was taken, as the level of DIF is likely to cancel out at the subscale level [39]. A series of t-tests performed on the person estimates from two subsets of items identified from principal component analysis of the residuals revealed that only 1.25% of cases had statistically significant t-values. The PSI was low (0.63) and the subscale was not well-targeted (average mean person location was −2.00, SD= 1.52).

In summary, to fit the original ‘Maladaptive’ 16 items to the Rasch model expectations it was necessary to: a) rescore all helplessness-hopelessness items and b) remove items 7, 16, 20, 25, and 29 as well as items 6, 9, 21, and 22. Hence, findings suggest that the remaining helplessness-hopelessness (rescored items 4, 12, 14, 15) and anxious preoccupation (original items 5, 13, 28) items can be combined to form a Maladaptive coping subscale.

## Discussion

This study aimed to substantiate the use of the mini-MAC with long-term cancer survivors, by investigating the structure of the scale using Rasch analysis. The psychometric properties of the original subscales of the mini-MAC were partially confirmed; however, item deletion and/or rescaling was necessary to some degree for all five subscales. It was also necessary to remove a substantial number of items from the combined adaptive and maladaptive subscales to achieve fit to the Rasch model.

At the item level, the anxious preoccupation, cognitive avoidance, fighting spirit, and helpless-hopelessness subscales [20] did not perform as expected and several items were deleted to achieve fit to the Rasch model expectations. Several of the deleted items were identified as problematic in previous studies. For example, Grassi et al., [21] found that the anxious preoccupation item 7 ‘*it is a devastating feeling’,* and the helplessness-hopelessness item 16 ‘*think it is the end of the world’*, loaded significantly onto more than one subscale and the fighting spirit item 18 ‘*I feel very optimistic’* loading was low (alpha < 0.4). Although these items were developed to capture coping strategies, at face value these may be seen to reflect outcomes rather than efforts to manage the demands of a stressful situation and it is unknown how they were interpreted by participants. On the other hand, the need to delete two of the four cognitive avoidance items (i.e., item 11 ‘*distract myself when thoughts about my illness come into my head’;* item 17 ‘*not thinking about it helps me cope’*) is inconsistent with previous research, which indicated that all these items satisfactorily contributed to one factor [20-23]. As these previous studies [20-23] have been conducted with individuals recently diagnosed with cancer and recruited from treatment centres (versus our long-term survivors recruited from a Registry) across a diversity of cultures (English, Italian, Chinese and Korean), the results of our study may reflect real differences in the construct of cognitive avoidance in cancer patients versus long-term cancer survivors.

Due to inconsistent endorsement of response categories, the helplessness-hopelessness, fatalism, and fighting spirit subscales were rescored. For fighting spirit and fatalism subscales, responses options 1=‘*definitely does not apply to me’* and 2=‘*does not apply to me’* were collapsed, whereas for the helplessness-hopelessness subscale, 3=‘*applies to me’* and 4=‘*definitely applies to me’* were collapsed. Issues with response categories can occur when the labelling of response options is ambiguous or too many response options are included. Given that careful clinical judgement was employed during the developmental phases of the MAC and mini-MAC [44], it is unlikely that the four-point response options are ambiguous; rather, it is possible that the mini-MAC has too many response options. Our findings are consistent with many studies undertaking Rasch analysis on other scales, which have found that respondents are not always able to distinguish between finer increments in responses options [34]. However, caution should be exercised in the interpretation of these findings as this is the first study to investigate the mini-MAC response format using Rasch analysis and these findings may be a function of the distinct study population and not the measure itself. In particular, for low scoring scales such as the Helplessness-Hopelessness subscale, it may be informative to further explore the relevance of these items to a population of long-term survivors.

Combining items to form the overarching subscales of *adaptive* and *maladaptive* coping, as proposed by Anagnostopoulos et al [24], was supported only after extensive changes to the already modified measure. Interestingly, the majority of fatalism items were deleted from the *adaptive* coping construct, despite many of these items seeming to express positive sentiments (i.e., item 8 ‘*I count my blessings’*, item 3 ‘*I put myself in the hands of God’*, item 19 ‘*I’ve had a good life, what’s left is a bonus’*). These findings are contrary to Watson et al’s [25] work with the MAC where items 3 and 19 contributed to the positive adjustment summary score and the remaining MAC fatalistic items loaded onto the negative adjustment summary score. Although one should be cautious comparing the MAC and mini-MAC, these results may suggest that fatalistic coping consists of both positive and negative characteristics and may not be a particularly good coping strategy for forcing into factors based on a-priori definitions of coping effectiveness. These results may also suggest that *‘adaptive’* is not the ideal label for this over-arching subscale. Indeed, this is supported by the fact that studies do not generally find significant positive correlations between adaptive coping subscales and mental health measures [23,24,29].

Examination of the remaining items within the *adaptive* and *maladaptive* subscales may support Anagopoulous et al’s [24] postulations that the *‘maladaptive’* subscale reflects cognitive and emotional representations of the illness (i.e., appraisal of the cancer), which in turn leads to using coping strategies measured by the *‘adaptive’* subscale. For example, feeling fearful (anxious preoccupation) or believing you are unable to cope (helplessness-hopelessness) may lead to avoidance, taking one day at a time, or a determination to fight. This postulation that anxious preoccupation and helplessness-hopelessness may reflect illness representations rather than coping strategies is also supported by findings from the MAC [21] studies that indicate anxious preoccupation coping and anxiety are highly correlated. Unfortunately we were unable explore this questions in the current study, and recommend that further research examine this issue.

## Conclusions

This study aimed to substantiate the use of the mini-MAC with long-term cancer survivors, and is the first study to undertake a Rasch analysis of the mini-MAC. Rasch analysis allowed a detailed assessment of the suitability of the response format, fit of each of the items, and the ability of the items to form a coherent set of subscales. We found that the psychometric properties of the original factor structure of the mini-MAC were partially confirmed. Some support was also found for the overarching subscales of *adaptive* and *maladaptive* coping; however, extensive modifications were necessary to achieve this result. Before a recommendation can be made about using this tool in a population of long term cancer survivors, further validation of these findings among other samples is necessary.

## Competing interests

The authors declare that they have no competing interests.

## Authors’ contributions

AZ participated in the conception of secondary research question, implementation the study protocol, interpretation of data and drafting of the manuscript. SL performed the statistical analysis; and participated in interpretation of the data and drafting of the manuscript. AB participated in study conception, design and acquisition of funding; conception of secondary research question, coordinated the implementation of the study protocol, and provided intellectual contribution to drafting the manuscript. JP participated in the statistical analysis and interpretation of the data, and provided intellectual contribution to drafting the manuscript. All authors read and approved the final manuscript.
